# 18 F-fluorothymidine uptake in follicular lymphoma and error-prone DNA repair

**DOI:** 10.1186/2191-219X-4-3

**Published:** 2014-01-08

**Authors:** Marielle J Wondergem, Ken Herrmann, Sergei Syrbu, Josée M Zijlstra, Nikie Hoetjes, Otto S Hoekstra, Saskia AGM Cillessen, Laura M Moesbergen, Andreas K Buck, Julie M Vose, Malik E Juweid

**Affiliations:** 1 Department of Haematology, VU University Medical Center (VUMC), De Boelelaan 1117, Amsterdam 1081 HV, The Netherlands; 2 Department of Nuclear Medicine, Technische Universität München (TUM), Munich 81675, Germany; 3 Department of Pathology, University of Iowa (UIHC), Iowa City, IA 52242, USA; 4 Department of Radiology and Nuclear Medicine, VU University Medical Centre (VUMC), Amsterdam 1081 HV, The Netherlands; 5 Department of Pathology, VU University Medical Centre (VUMC), Amsterdam 1081 HV, The Netherlands; 6 Division of Haematology/Oncology, Nebraska Medical Center, Omaha NE 68198, USA; 7 Department of Radiology Nuclear Medicine, University of Jordan, Amman 11953, Jordan; 8 Department of Nuclear Medicine, Universitätsklinikum Würzburg, Würzburg 97080, Germany

**Keywords:** 18 F-fluorothymidine uptake, Positron emission tomography, Follicular lymphoma, Non-Hodgkin’s lymphoma, DNA repair

## Abstract

**Background:**

We observed a disproportional 18 F-fluorothymidine (F-FLT) uptake in follicular lymphoma (FL) relative to its low cell proliferation. We tested the hypothesis that the ‘excess’ uptake of 18 F-FLT in FL is related to error-prone DNA repair and investigated whether this also contributes to 18 F-FLT uptake in diffuse large B cell lymphoma (DLBCL).

**Methods:**

We performed immunohistochemical stainings to assess the pure DNA replication marker MIB-1 as well as markers of both DNA replication and repair like PCNA, TK-1 and RPA1 on lymph node biopsies of 27 FLs and 35 DLBCLs. In 7 FL and 15 DLBCL patients, 18 F-FLT-PET had been performed.

**Results:**

18 F-FLT uptake was lower in FL than in DLBCL (median SUVmax 5.7 vs. 8.9, *p* = 0,004), but the ratio of 18 F-FLT-SUVmax to percentage of MIB-1 positive cells was significantly higher in FL compared with DLBCL (*p* = 0.001). The median percentage of MIB-1 positive cells was 10% (range, 10% to 20%) in FL and 70% (40% to 80%) in DLBCL. In contrast, the median percentages of PCNA, TK-1 and RPA1 positive cells were 90% (range, 80 to 100), 90% (80 to 100) and 100% (80 to 100) in FL versus 90% (60 to 100), 90% (60 to 100) and 100% (80 to 100) in DLBCL, respectively.

**Conclusions:**

This is the first demonstration of a striking discordance between 18 F-FLT uptake in FL and tumour cell proliferation. High expression of DNA replication and repair markers compared with the pure proliferation marker MIB-1 in FL suggests that this discordance might be due to error-prone DNA repair. While DNA repair-related 18 F-FLT uptake considerably contributes to 18 F-FLT uptake in FL, its contribution to 18 F-FLT uptake in highly proliferative DLBCL is small. This apparently high contribution of DNA repair to the 18 F-FLT signal in FL may hamper studies where 18 F-FLT is used to assess response to cytostatic therapy or to distinguish between FL and transformed lymphoma.

## Background

Positron emission tomography (PET) with 18 F-fluorodeoxyglucose (18 F-FDG) is widely used in (re)staging and therapy monitoring of lymphoma. Uptake of 18 F-FDG in malignant cells is only partially related to proliferation [1,2]. 18 F-fluorothymidine (18 F-FLT), a thymidine analogue, has been tested for its ability to more specifically measured tumour proliferative activity [3]. 18 F-FLT uptake is determined by thymidine kinase-I (TK-1) activity, an enzyme closely linked to DNA synthesis and, hence, indirectly to tumour cell proliferation [4].

Non-Hodgkin’s lymphomas can have low and high proliferative activity, e.g. indolent lymphomas such as low-grade FL, and aggressive lymphomas, such as diffuse large B cell lymphoma (DLBCL), respectively. Both can be imaged by PET, using either 18 F-FDG or 18 F-FLT. Previously, a close correlation was reported between 18 F-FLT uptake and the proliferation fraction in a mixed population of patients with FL and DLBCLs [5].

We recently reported that MIB-1, an antibody specific for the pure proliferation/replication marker Ki67, expectedly demonstrated a small percentage of positive cells in FL and a high percentage in DLBCL. However, when analyzing 18 F-FLT uptake in FL and DLBCLs, we observed that uptake of 18 F-FLT is disproportional to cellular proliferation in untreated FL; the uptake is much higher than expected based on cellular proliferation alone [6].

Since the uptake of thymidine is not only increased in replicative DNA synthesis in proliferating cells but also with enhanced DNA repair in quiescent cells, we now aimed to test the hypothesis that the ‘excess’ uptake of 18 F-FLT in FL may be related to error-prone DNA repair known to occur in FL and to be responsible for the generation of somatic hypermutation (SHM) and class-switch recombination (CSR). In addition, we investigated whether this DNA repair contributes to 18 F-FLT uptake in DLBCLs since SHM/CSR also occurs in most DLBCLs.

## Methods

Twenty-two patients with lymphoma (7 FL and 15 DLBCL) underwent whole-body 18 F-FLT-PET prior to treatment in prospective clinical trials at VU University Medical Center (NTR 4187(Dutch trial registry)) and Technische Universität München (TUM Local ethics committee nr 978/03) [7,8]. In these patients, paraffin sections of lymph node biopsies obtained prior to imaging were retrieved for immunohistochemical staining. In addition, at the University of Iowa Hospitals and Clinics (UIHC), paraffin sections of 20 cases of FL and 20 cases of DLBCL presenting between 1990 and 2008 were randomly selected from the files of the Department of Pathology. Diagnosis was revised according to the WHO classification 2008. All biopsies had been obtained prior to treatment for diagnostic reasons, and all FL were grade I or II.

### Immunohistochemistry

Immunohistochemistry on all samples was performed at the Department of Pathology of UIHC. Sections of the formalin-fixed, paraffin-embedded tissue were deparaffinized in xylene, rehydrated in graded alcohols and rinsed. After antigen retrieval, endogenous peroxidase activity was blocked using 3% H_2_O_2_. The sections were then incubated with antibodies against Ki-67 (Clone MIB-1, DakoCytomation; Carpinteria, CA, USA), which is specific only for replicative DNA synthesis (proliferation) as well as for the replication and repair markers proliferating cell nuclear antigen(PCNA) and replication protein A (RPA32/RPA2) (Abcam Inc., Cambridge, MA, USA), and TK1 (Clone F12, Novus Biologicals, Inc., Littleton, CO, USA), for 30 to 60 min at room temperature. For the detection of bound antibodies, the EnVision technique (DakoCytomation; Carpinteria, CA, USA) with diaminobenzidine as a substrate was used. The percentage of cells positive for nuclear expression of MIB-1, PCNA, RPA32/RPA2 and TK1 in follicles and diffuse areas were counted in 5 × 400 random fields and an average was calculated [9]. To include DNA synthesis for both proliferation and repair, all positive cells were counted independent of staining intensity for both PCNA and TK-1 [9].

### 18 F-FLT PET

At VUMC, PET imaging was done using a full ring PET-CT scanner (Philips Gemini TF64). Acquisitions (3 min/bed) started approximately 60 min after about 185 MBq 18 F-FLT iv, covering a skull mid-thigh trajectory. At TUM, a full ring PET scanner (ECAT HR+, Siemens/CTI) was used, acquisition starting 45 min after approximately 300 MBq 18 F-FLT iv. Circular regions of interest were drawn semi-automatically containing the area of increased 18 F-FLT uptake to calculate standardized uptake values (SUV) as described before [7,10]. Maximum values of 18 F-FLT-SUV were calculated for all biopsied lesions, all larger than 3 cc, minimizing partial volume effects.

### Statistics

18 F-FLT SUVmax and the 18 F-FLT SUVmax to MIB-1 ratio were compared using the Mann–Whitney U test. Correlation coefficients were calculated using the Spearman’s rho test. Differences were considered significant at a level of *p* < 0.05. The model was fitted using linear regression analysis.

## Results

In the 22 patients who underwent 18 F-FLT imaging, 18 F-FLT uptake was lower in FL than in DLBCL (median SUVmax 5.7 (range 3.0 to 6.7) versus 8.9 (range 3.7 to 18; *p* = 0.004)), consistent with the lower proliferation rate of FL compared with DLBCL (median percentage of MIB-1 positive cells 10% in the 7 FL patients and 70% in the 15 DLBCL patients (Table 1)). However, in FL 18 F-FLT-SUVmax relative to tumour, cell proliferation was disproportionally high compared to that of the DLBCL patients (mean ratio of 0.38 for FL vs. 0.14 for DLBCL, *p* = 0.001) (Table 1). While the median SUVmax of FL was 64% of that in DLBCL, the median percentage ofMIB-1 positive cells of FL was only 14% of that in DLBCL.

**Table 1:** Immunohistochemical values and 18 F-FLT uptake (SUV max) for all patients who underwent PET scanning and biopsy

	**Lymphomatype**	**MIB1 (%)**	**PCNA (%)**	**TK1 (%)**	**RPA (%)**	**18 F-FLT (SUVmax)**	**18 F-FLT/MIB ratio**
1	Follicular	10	100	70	80	6.2	0.62
2	Follicular	15	100	80	100	3.6	0.24
3	Follicular	20	90	80	100	3.0	0.15
4	Follicular	10	80	100	100	4.7	0.47
5	Follicular	20	80	100	ND	6.7	0.34
6	Follicular	20	80	100	100	6.5	0.33
7	Follicular	10	95	ND	ND	5.7	0.57
8	Transformed	70	100	80	100	8.1	0.11
9	Transformed	50	100	90	100	5.4	0.11
10	Transformed	80	80	100	80	9.8	0.12
11	Transformed	70	70	80	100	6.1	0.08
12	Transformed	80	90	100	100	14.5	0.18
13	DLBCL	60	90	90	ND	10.5	0.18
14	DLBCL	70	70	60	ND	8.4	0.12
15	DLBCL	60	60	80	ND	9.8	0.16
16	DLBCL	80	70	90	ND	18.0	0.23
17	DLBCL	60	90	90	ND	8.9	0.15
18	DLBCL	40	60	90	ND	3.7	0.09
19	DLBCL	70	90	100	ND	7.2	0.10
20	DLBCL	70	90	100	ND	13.3	0.19
21	DLBCL	40	90	70	ND	8.4	0.21
22	DLBCL	80	80	80	ND	9.6	0.12

ND, not done.

In order to determine whether the disproportional increase of 18 F-FLT uptake in FL relative to its low cell proliferation might be related to error-prone DNA repair known to occur in FL, we stained tumour samples of the 22 patients for the DNA replication and repair markers PCNA, TK-1 and RPA1 in addition to the pure DNA replication marker MIB-1. As shown in Table 1, the median percentage of MIB-1 positive cells was 10% (range, 10% to 20%) in FL and 70% (40% to 80%) in DLBCL. In contrast, the median percentages of PCNA, TK-1 and RPA1 positive cells were 90% (range, 80 to 100), 90% (80 to 100) and 100% (80 to 100) in FL versus 90% (60 to 100), 90% (60 to 100) and 100% (80 to 100) in DLBCL, respectively.

The immunostaining results of the two patient groups with and without 18 F-FLT PET (22 and 40 (20 FL and 20 DLBCL), respectively) were very similar (Table 2); again in the group without 18 F-FLT PET, the median percentage of MIB-1 positive cells was 10% (range, 5% to 30%) in FL and 80% (60%to 90%) in DLBCL. And also, the median percentage PCNA, TK-1 and RPA positive cells were 90% (range 80 to 100), 95% (90 to 100) and 100% in FL versus 100% (80 to 100), 95% (70 to 100) and 100% in DLBCL, respectively.

**Table 2:** Immunohistochemical values for all patients with biopsy material only

	**Follicular**				**DLBCL**			
	**MIB1 (%)**	**PCNA (%)**	**TK1 (%)**	**RPA (%)**	**MIB1 (%)**	**PCNA (%)**	**TK1 (%)**	**RPA (%)**
1	5	90	95	ND	80	100	100	100
2	20	90	95	100	80	100	80	100
3	10	100	95	100	80	100	70	100
4	10	100	95	ND	60	100	100	100
5	10	90	90	ND	80	100	95	100
6	10	80	100	100	80	100	95	100
7	10	90	95	100	60	80	95	100
8	10	100	90	100	70	100	100	ND
9	20	90	95	100	80	100	90	ND
10	20	90	90	ND	60	80	90	100
11	10	100	100	100	60	80	70	ND
12	10	90	95	ND	90	100	100	100
13	10	90	95	ND	90	100	100	100
14	10	100	100	100	80	90	95	100
15	10	100	95	ND	80	100	90	100
16	20	100	95	ND	90	100	95	100
17	5	90	100	100	60	90	95	100
18	10	90	95	100	90	100	80	ND
19	30	80	90	100	70	100	100	100
20	10	100	95	ND	70	100	100	100

**Figure 1:**
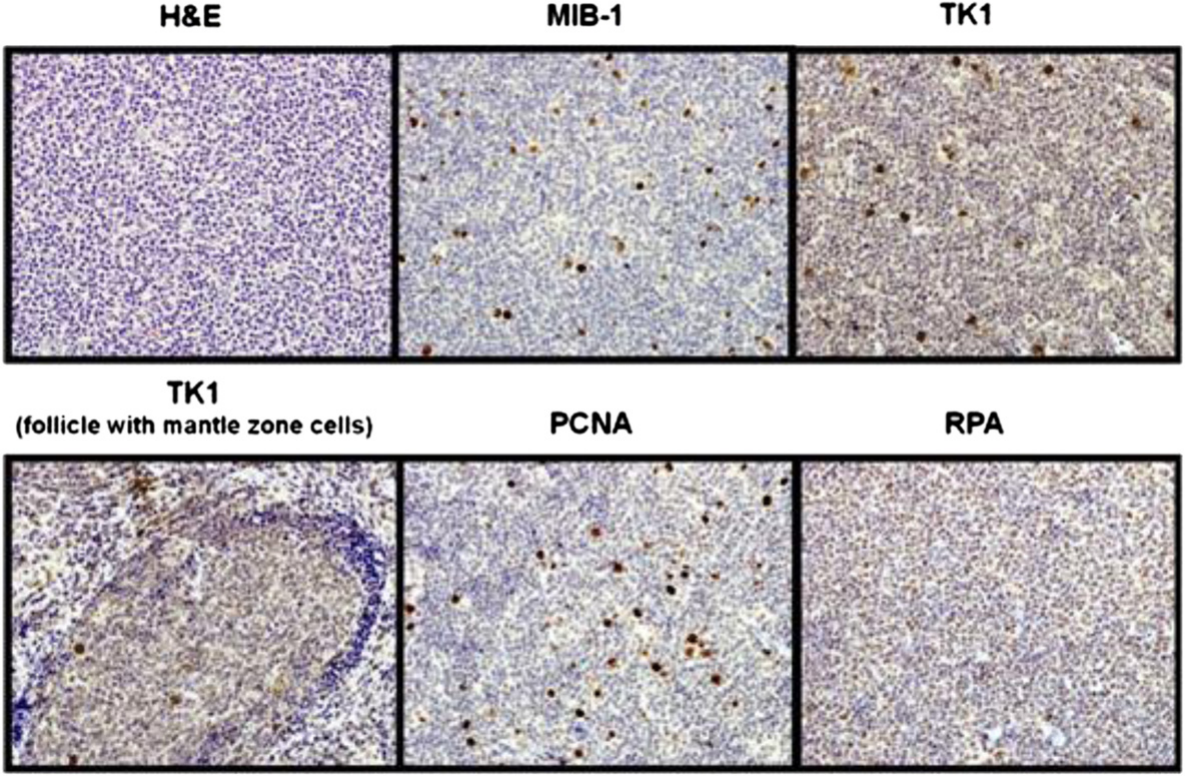
**H and E, MIB-1, TK-1, PCNA and RPA staining in an untreated patient with grade 1 FL.** Estimated percentage ofMIB-1, TK-1, PCNA- and RPA-positive cells is 5%, 100%, 90%, and 100%, respectively. Note the specific TK-1 staining of germinal centre cells with lack of TK-1 staining of bystander mantle zone cells.

Both in FL and DLBCL, PCNA and TK-1 showed a characteristic staining pattern with 3+ or 4+ in proliferating cells and 1+ to 2+ staining of quiescent cells (Figure 1). The intensity of PCNA, TK-1 and RPA1 staining in quiescent or proliferating cells was similar in FL and DLBCL.

**Figure 2:**
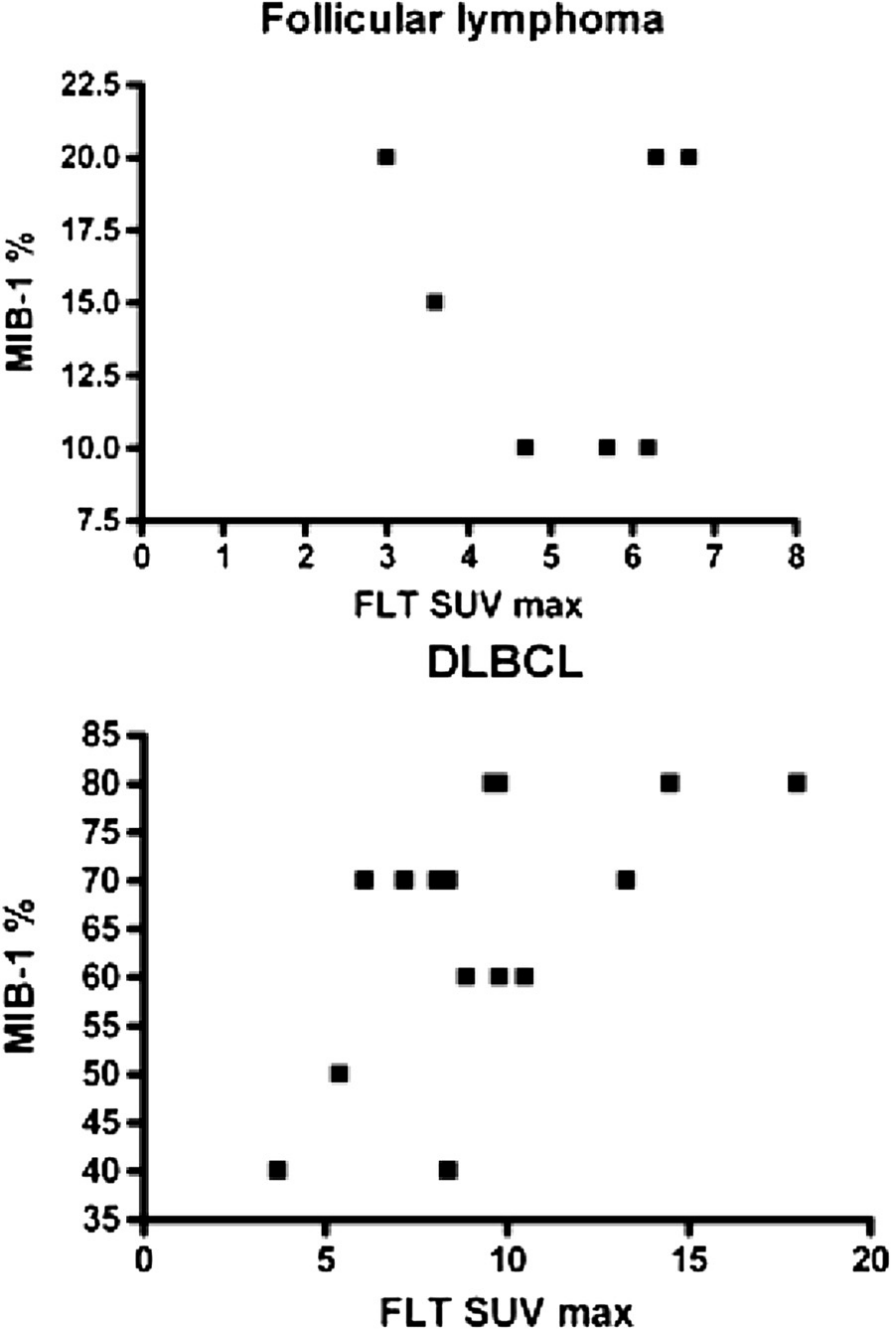
**Correlation between 18 F-FLT uptake and percentage MIB-1 positive cells.** Absent correlation between 18 F-FLT uptake and the percentage of MIB-1 positive cells in FL (*r* = 0.15, *p* = 0.74) and a significant correlation in DLBCL and transformed lymphoma (*r* = 0.55, *p* = 0.03).

There was a statistically significant positive correlation between 18 F-FLT uptake and the percentage of MIB-1 positive cells for DLBCL (*r* = 0.55, *p* = 0.03) but not for FL (*r* = 0.15, *p* = 0.74; Figure 2).

We found no significant correlation between FLT uptake and%TK-1 positive cells when cell staining for TK1 was considered positive regardless of staining intensity (*r* = 0.44, *p* = 0.18). Only if those cells with 3+ or 4+ staining intensity were considered positive (i.e. the proliferating cells, which are the same cells that are MIB-1 positive), was there a significant correlation, mirroring the correlation between FLT uptake and MIB-1 expression.

## Discussion

The unexpected high uptake of 18 F-FLT in FL relative to its cell proliferation reflected by the higher 18 F-FLT- SUVmax to MIB-1 ratio in FL compared to DLBCL is in line with a previous study, where the ratio of 3H-thymidine uptake to percentage of MIB-1-positive cells in FL was 1.5 times that in DLBCL, associated with relatively increased expression of DNA repair proteins (PCNA) [11]. The disproportionally high expression of DNA replication and repair markers (TK-1, PCNA, RPA) compared with the specific replication marker MIB-1 suggests that the increase of 18 F-FLT uptake in FL might be due to DNA repair in quiescent (and proliferating) FL cells involved in error-prone DNA repair known to occur in the germinal centres of FL. This error-prone repair is responsible for generation of SHM and CSR, which constitute one of the bases for our innate immunity. In fact, both PCNA and RPA have been reported to play a role in SHM/CSR [11]. The hypothesis that DNA repair might contribute to total 18 F-FLT uptake can be supported by observations in animal models; De Saint-Hubert et al. showed an accumulation of cells in S-phase 2days after cyclophosphamide treatment of mice with Burkitt’s lymphoma, suggesting repair of the chemotherapy-induced DNA cross-links, accompanied by a later decrease of 18 F-FLT uptake, at 7 days posttreatment [12].

We found high percentages of positive cells for both FL and DLBCL when looking at PCNA, TK-1 and RPA1, all known to be involved in both DNA replication and repair [13,14]. Others also found high levels of PCNA positivity in DLBCLs [15] and similar levels of TK-1 activity in low- and high-grade lymphoma [16]. Chang et al. show high PCNA positivity in FLs with positivity in both follicular and interfollicular areas without specifying the staining intensity [17]. The characteristic staining pattern of PCNA and TK-1 seen in both FL and DLBCL with 3+ or 4+ in proliferating (i.e. MIB-1 positive) cells and 1+ to 2+ staining of quiescent cells is explained by the fact that both PCNA and TK-1 show a striking increase in the expression during S-phase but are also expressed albeit to a lesser extent in other phases of the cell cycle [13,18]. In fact, PCNA can be detected at higher levels in all phases of the cell cycle after cell damage by radiation, suggesting DNA repair [13,19]. High expression of DNA replication and repair markers (TK-1, PCNA, RPA) in FL was not only observed in the 7 FL patients who underwent 18 F-FLT PET but was confirmed in an additional 20 patients with FL in whom only immunohistochemical studies were performed. The same staining intensity and pattern observed in the 15 patients with DLBCL who underwent FLT-PET imaging was confirmed in the additional 20 DLBCL patients with immunohistochemical studies only.

To illustrate the potential relative contribution of DNA repair synthesis to 18 F-FLT uptake in FL and DLBCL, a model was fitted using linear regression on the data depicted in Table 1. Since the FLT uptake essentially reflects the TK1 activity, in this model, we assumed the TK-1 activity per repairing quiescent cell to be a third of that in a proliferating cell, compatible with the 1+ to 2+ versus 3+ to 4+ staining intensity of quiescent versus proliferating cells.

$$\begin{array}{l} \text{SUVmax}=-1.4+\left(0.15\times \%\text{proliferating}\;\text{cells}\right)\\ \;+\left(0.05\times \%\text{quiescent}\;\text{cells}\right) \end{array}$$

% proliferating cells = %MIB -1 positive cells.

% quiescent cells = % TK-1 positive cells -%MIB-1 positive cells.

The model assumes that TK-1 activity per proliferating cell is similar in FL and DLBCL. Since no difference in S-phase duration between FL and DLBCL is reported and DNA synthesis rate (and hence TK-1 activity) is proportional to S-phase duration, this assumption seems valid [20]. R-square is 0.51, indicating a moderate fit, an interesting result considering the relatively small number of patients who underwent PET scans with FLT.

While SHM/CSR also occurs in most DLBCLs (explaining the discordance between percentage of MIB-1-positive and TK-1-positive cells in most DLBCL in our study), the relative contribution of DNA repair to 18 F-FLT uptake in DLBCL is apparently small in most cases, so that a significant correlation between percentage of MIB-1-positive cells and 18 F-FLT uptake can be found. The absence of this correlation in FL might be due to the increased DNA repair, interfering with the correlation, or the small number of samples. Consequently, this should be confirmed in a larger sample, also including higher grade FLs, with possible higher MIB-1 percentages.

A difference between the composition of a DLBCL and a FL is the presence of a microenvironment in FL. To determine the percentage positive cells for every immunohistochemical marker, we counted larger areas of the FL, including both the lymphoma cells and the microenvironment. It reflects the fact that 18-FLT uptake in the whole lymph node is caused by uptake in both lymphoma- and microenvironmental cells. This ‘average expression’ method was reported by Chalkidou et al. to give the best results for correlation of proliferation markers and 18 F-FLT uptake [21]. However, we cannot determine which proportion of the uptake of 18 F-FLT in FL (caused by proliferation or repair) is explained by uptake in the microenvironmental cells. The fact that the composition of the microenvironment can vary considerably in its proportions of T cells, macrophages and follicular dendritic cells between FLs, all with different unknown contributions to total 18 F-FLT uptake is an additional complicating factor in hypothesis generation [17].

Imaging with PET has been used in an attempt to distinguish indolent from transformed lymphoma. Since 18 F-FDG PET scans have shown considerable overlap in SUV between FL and transformed lymphoma, and since the main characteristic of transformation is increased proliferation, imaging with 18 F-FLT was also investigated. Unexpectedly, 18 F-FLT showed similar overlap as 18 F-FDG in SUV of FL and transformed lymphoma [22-25]. In our FL and transformed lymphoma patients, we also found overlap; SUVmax in FL was 3.0 to 6.7, in transformed lymphoma 5.4 to 14.5. This might, at least in part, be explained by our findings of additional DNA repair-related 18 F-FLT uptake in non-proliferating FL cells or the microenvironment.

18 F-FLT has also been used to image a decrease in proliferation following effective cytostatic therapy, thus predicting response [8,26]. However, if 18 F-FLT also images DNA repair in addition to proliferation in FL, the change in 18 F-FLT uptake following FL treatment will be confounded by the high contribution of DNA repair and, hence, significant changes in cellular proliferation may be missed or obscured. For example, if the pretreatment FL SUVmax is 5.0 with only 1.0 SUV unit related to proliferation with the remaining 4.0 SUV units actually related to repair, a 50% decrease in proliferation without any change in DNA repair will change the SUV from 5.0 to only 4.5, an insignificant change erroneously indicating lack of anti-proliferative effect. Cytotoxic therapy might even enhance DNA repair. Fortunately, it appears that the increased 18 F-FLT uptake that is due to increased DNA repair following cytotoxic therapy (i.e., gemcitabine) is only transient, subsiding within 48 h after dose administration, although this phenomenon has been described to be dependent on the cytostatic drugs that are used [27,28]. Following cytostatic therapy, accumulation of cells in S phase has been described, increasing 18 F-FLT uptake [12,29]. Thus, delaying 18 F-FLT imaging by at least 48 h or more after treatment to assess response (depending on the cytostatic agent and its mechanism of action) may be sufficient to overcome this part of the problem. However, further research is needed to validate this hypothesis.

## Conclusions

To our knowledge, this is the first demonstration of a striking discordance between 18 F-FLT uptake in FL and tumour cell proliferation. Our immunohistochemical finding of high expression of DNA replication and repair markers in FL suggests that the disproportional increase of 18 F-FLT uptake might be due to error-prone DNA repair, responsible for SHM/CSR in FL cells or the microenvironment. This may hamper studies where 18 F-FLT is used for assessing response to cytostatic therapy or to distinguish between FL and transformed lymphoma. Further research is needed to elucidate the mechanism of 18-F-FLT uptake to be able to accurately interpret changes in uptake following therapy or histologic transformation.

## Competing interests

The authors declare that they have no competing interest.

## Authors’ contributions

MW, KH, SS, JZ, OH, SC, AB, JV and MJ wrote the paper. MW, JV, AB and MJ designed the research. KH and SC provided patient data. SS, SC and LM performed the research. AB, NH and OH analyzed the scans. MW and MJ analyzed and interpreted the data. All authors read and approved the final manuscript.
